# Disease-specific quality of life in patients with diabetic neuropathy

**DOI:** 10.15537/smj.2022.43.4.20210861

**Published:** 2022-04

**Authors:** Mushabab Alghamdi, Lukman F. Owolabi, Bappa Adamu, Magaji G. Taura, Abubakar Jibo, Mohammed Almansour, Saeed N. Alaklabi, Mohammed A. Alghamdi, Isa A. Imam, Reda Abdelrazak, Ahmad Rafaat, Muktar H. Aliyu

**Affiliations:** *From the Departmentof Internal Medicine (Alghamdi M, Owolabi, Adamu, Taura, Jibo, Alaklabi, Alghamdi MA, Imam, Abdelrazak), University of Bisha Medical College; from the Departmentof Internal Medicine(Rafaat), King Abdullah Hospital, Bisha; from the Departmentof Internal Medicine (Almansour), King Fahad Medical City, Riyadh; from the Department of Health Policy (Aliyu), Vanderbilt University Medical Center, Nashville, United States of America.*

**Keywords:** quality of life, diabetes melitus, disease-specific

## Abstract

**Objectives:**

To compare health-related quality of life (HRQoL) among patients with diabetes mellitus (DM) and diabetic neuropathy (DN) (D+N) with patients with DM without DN (D-DN) and healthy participants. To evaluate factors associated with poor HRQoL in patients with DN.

**Methods:**

This study included 306 participants residing in Bisha, Saudi Arabia. Patients with DM were screened for DN using the Michigan Neuropathy Screening Instrument. Neuropathy severity, disability and HRQoL were determined using the Neuropathy Severity Scale (NSS), the Neuropathy Disability Score (NDS), and the Norfolk Quality of Life-Diabetic Neuropathy (QOL-DN) tool, respectively. Nerve conduction studies (NCSs) were also performed.

**Results:**

The D+DN group had poorer overall and domain HRQoL scores compared to the D-DN group (*p*<0.001). There was a strong correlation between overall HRQoL score and both NDS and NSS scores in the D+DN group (ρ= −0.71 and *p*<0.0001; ρ= −0.81 and *p*<0.0001, respectively). There was also a significant difference in all mean HRQoL domain scores between D+DN participants with normal and abnormal NCS. Physical inactivity (*p*=0.043), duration of DM (*p*<0.0001), abnormal NCS, NSS (*p*<0.0001), and NDS (*p*<0.0001) predicted HRQoL in the D+DN group.

**Conclusion:**

D+DN participants had a worse HRQoL compared with D-DN and healthy counterparts. NDS, NNS, physical inactivity, abnormal NCS, and duration of DM independently predicted poor HRQoL in D+DN participants.

**D**iabetes mellitus (DM) is one of the most prevalent chronic diseases and is a growing global health concern. Worldwide, it is expected that the number of people living with DM will increase dramatically, reaching more than 500 million by 2035.^1^ The tremendous increase in the prevalence of this disease will be accompanied by an increase in chronic diabetic microvascular and macrovascular complications.^2^ Diabetic neuropathy (DN), one of the most prevalent complications of DM, significantly impacts the quality of life and increases the risk of early death in patients with DM.^2^

Diabetic neuropathy, a microvascular complication of DM, is attributed to chronic hyperglycemia and is described as the presence of peripheral nerve dysfunction in a patient with DM after exclusion of other causes. The heterogeneous sequelae of DM affect different parts of the nervous system and cause diverse clinical manifestations.^3^ Diabetic neuropathy may cause secondary complications, such as Charcot arthropathy, foot ulcer, and lower limb amputation.^4^ Diabetic neuropathy often has an insidious onset and is asymptomatic in approximately 50% and symptomatic with sensory manifestations in approximately 20% of affected individuals.^5^ Symptoms of DN may include pain, paresthesia, numbness, hyperesthesia, and gait imbalance, with the potential for foot ulcer and amputation.

The evaluation of health-related quality of life (HRQoL) is increasingly valued as a vital arm of the assessment of chronic diseases and their complications.^6^ The existing evidence has revealed a negative impact of DM and its complications, including DN, on people’s HRQoL and psychosocial and physical well-being.^7^

Previous studies have reported that changes in the physical domain of HRQoL in patients with DN are associated with patient’s age and body mass index (BMI). Poor mental HRQoL has been associated with female gender, smoking, duration of DM, and BMI. The poor HRQoL observed in patients with DN has been attributed to factors such as pain, mood, unhealthy mental state, disturbed sleep, and impaired daily activities^.8,9^

The need to be cognizant of the broad impact of DN in patients with DM beyond the specific disease process and the importance of assessing patient-reported outcomes such as HRQoL have been increasingly recognized in medical practice.

In addition to the impact that poor HRQoL has on the well-being of patients and their relatives, it plays a role in how successful the medical management of DM and its complications will be; thus, an assessment of HRQoL in every patient with DM and DN is necessary.

Unlike other DM complications, the impact of DN on HRQoL has not been extensively studied using a disease-specific HRQoL tool, particularly in Saudi Arabia, where DM is prevalent.^10^ Data on the HRQoL of patients with DN are critical in DN treatment centers to optimize the surveying of perceived health problems, screening and monitoring of psychosocial issues, and performance of outcome measures, medical audits, and cost-utility analyses.^6^

We hypothesized that there would be notable differences in HRQoL, as measured by a disease-specific HRQoL tool, between DM patients with DN (D+DN), DM patients without DN (D-DN), and healthy participants.

The study aimed to compare HRQoL in a cohort of Saudi patients comprising D+DN, D-DN, and healthy participants to evaluate the factors associated with poor HRQoL in patients with D+DN.

## Methods

This cross-sectional study included 306 participants recruited from the diabetes, endocrinology, and metabolic diseases center of a medical clinic, medical outpatient, and electrophysiology laboratory from December to June 2021 at King Abdullah Hospital (KAH), Bisha, Saudi Arabia.

The required sample size was calculated using the STATA software package (STATA, Inc., version 16, Texas 77845-4512. USA) with the following requisite parameters: statistical power (α) = 0.8, statistical significance (α) = 0.05, effect size = 5 value points, and expected standard deviation (SD =10 points using a balanced study design. The minimum sample size was determined to be 92 subjects per group. In order to reduce the influence of unknown variables on the sample size calculation, the sample size was increased to 102 participants in each group.

The participants were consecutively recruited and included 102 patients with DM (type I and II) and DN, 102 patients with DM (type I and II) without DN (based on the presence or absence of peripheral neuropathy during screening), and 102 apparently healthy controls from the general population.

The inclusion criteria were all participants were adult (>18 years of age) and were matched for age and gender using nearest neighbor matching method.^11^ Participants were excluded from the study if they had a mental or cognitive illness, were hospitalized at the time of the study, or were undergoing treatment for cancer.

Screening for DN was performed using the Michigan Neuropathy Screening Instrument (MNSI), which is widely used for the evaluation of distal symmetrical peripheral neuropathy in DM. Patients with abnormal test results, defined as ≥4 abnormal items on the MNSI questionnaire, were considered to have DN and were included in the D+DN group.^12^ The severity of neuropathy was determined in the D+DN group using the Neuropathy Severity Scale (NSS), which evaluates neuropathic symptoms such as burning, cramping, aching, fatigue, tingling sensations or numbness, and nocturnal exacerbation. Disability due to neuropathy was assessed using the Neuropathy Disability Score (NDS), which comprises measurements of ankle reflex, temperature, pinprick sensation, vibration sense tested with a 128-Hz tuning fork, and monofilament test. A detailed medical history, followed by somatic and neurological status, was obtained from all participants. While obtaining medical history, we specifically focused on demographic data and comorbidities that were important for this study.

The participants’ medical records were also reviewed for the presence of comorbidities, DM-related complications, lipid profiles, hemoglobin A1c (HbA1c), sociodemographic characteristics, duration of DM in years, medication prescription, and drug adherence. The variables of interest were derived from a literature search. Search for relevant previous studies was performed using related MESH terminologies and relevant search engines (PubMed, Google, Google scholar, and Endnote). A disease-specific HRQoL tool was administered to all participants. The study was carried out in Bisha, KSA between November 2020 and June 2021.

### Measuring health-related quality of life

Health-related quality of life was measured using the Norfolk Quality of Life-Diabetic Neuropathy (Norfolk QOL-DN) instrument, which has been established as a reliable outcome measure across multiple populations and is sensitive to both small- and large-fiber abnormalities.^12^ The Norfolk QoL-DN instrument is a comprehensive and validated 47-item questionnaire designed to capture the entire spectrum of DN related to small fiber, large fiber, and autonomic neuropathy.^13^ It comprises 2 sets of questions: queries related to symptoms experienced by the diabetic patient and those related to the impact of the diabetic patient’s neuropathy on activities of daily living (ADLs). The questions in the instrument are categorized into 6 exploratory domains, including total quality of life, symptoms, ADLs, physical functioning/large fiber, small fiber, and autonomic neuropathy.^5,14^ The Norfolk QOL-DN has a fairly good reliability profile, with a Cronbach alpha of 0.60–0.80 for all 3 clinical groups, 0.74-0.86 for patients with DM and DN, 0.63-0.80 for patients with DM without DN, and 0.62-0.79 for healthy controls.^5^

Regarding the Norfolk QOL-DN scoring algorithm adopted in this study, all items in the symptom domain (items 1-7) were assigned a score of either 1 or 0, indicating the presence or absence of the indicated symptoms. With the exception of items 31 and 32, the other items were scored based on a 5-point Likert scale (0-4, “No Problem” to “Severe Problem”). For item 31, “Poor” was assigned a score of 2, “Fair” a score of 1, “Good” a score of 0, “Very Good” a score of –1, and “Excellent” a score of –2. Item 32 was also scored on a scale of −2 to 2, with −2 indicating “Much Better,” −1 indicating “Somewhat Better,” 0 indicating “About the Same,” 1 indicating “Somewhat Worse,” and 2 indicating “Much Worse.” The algorithm used for the summation of scores was as follows: total quality of life, Σ (1-7, 8-35); physical functioning/large fiber, Σ (8, 11, 13-15, 24, 27-35); ADLs, Σ (12, 22, 23, 25, 26); symptoms, Σ (1-7, 9); small fiber, Σ (10, 16, 17, 18); and autonomic, Σ (19, 20, 21). Scores were calculated without weighting and were reported as the sum of the integers of the listed questionnaire items.^15^ A higher score on the Norfolk QOL-DN denotes a poorer quality of life.

### Electrophysiological study

Nerve conduction studies (NCS) were conducted on consenting patients in the D+DN group using the Natus Nicolet Viking Quest electromyography machine. Median (mixed), ulnar (mixed), and radial nerves in the upper limbs and tibial (motor), peroneal (motor), superficial peroneal (sensory), and sural (sensory) nerves in the lower limbs were assessed. An abnormal NCS result was determined based on local NCS reference values in the electromyography laboratory.

### Other definitions

Diabetes mellitus: diabetes mellitus was defined as the fulfillment of one of the following conditions: a) a self‐reported diagnosis by a health professional, b) the use of glucose‐lowering medications, and c) HbA1c >6.5% or fasting blood glucose ≥ 126 mg/dL.^16^

Diabetic neuropathy (outcome variable): diabetic neuropathy was defined as a score of >4 on the MNSI questionnaire and confirmed with electrophysiological evidence of neuropathy on NCS.^12^

Dyslipidemia (covariate): dyslipidemia was defined as the presence of one of the following: total cholesterol ≥5.2 mmol/L, high-density lipoprotein ≤1.3 mmol/L, low-density lipoprotein ≥3.4 mmol/L, or triglycerides ≥1.7 mmol/L based on the National Cholesterol Education Program Adult Treatment Panel III guidelines, or current use of cholesterol-lowering drugs.^17^

Sociodemographic characteristics (covariates): Gender was dichotomized as male or female. Level of education was classified as educated (formal Western or Islamic) and not educated.

Lifestyle characteristics (covariates): History of stroke or transient ischemic attack (TIA) was defined as any self-reported participant history of stroke or TIA. Physical inactivity was based on the average number of hours of physical activity performed per day (including working and leisure activities), and classified as either “no” if respondents were not involved in moderate or strenuous exercise for ≥4 hours per week, or “yes” for all other quantities of physical activity.^18^

Smoking status implied tobacco use and was classified as “never” (if the participant had never used any type of tobacco product) and “ever” (if the participant smoked cigarettes or used any type of tobacco product in the past 12 months).^15^ Alcohol use was defined as “never” when the participant had never consumed any form of alcoholic drink and “ever” when the participant had consumed any form of alcoholic drink previously or in the last 30 days.^19^

Anthropometric measurements: measurement of height (cm) and weight (kg) was performed using a standard standiometer and weight scale, respectively, and used to calculate BMI (weight in kg divided by the square of height in meters) based on the World Health Organization guidelines.^20^

The study was approved by the ethics committee of the University of Bisha Medical College (UBCOM-RELOC Reg No: H-06-BH-087). The study was carried out in accordance with the principles of the Helsinki declaration.

### Data analysis

Data analysis was performed using the STATA software package (STATA, Inc., version 16, College Station, TX: StataCorp LLC). We assessed the missingness of missing-at-random data for HRQoL and other variables by eliminating them using the deletion method. Descriptive statistics were expressed as mean±SD in the case of parametric data, such as age, and median with interquartile range (IQR) for non-parametric data, such as NSS and NDS scores. Categorical data, such as sociodemographic characteristics, drug compliance, illness history, symptoms, and NCS findings, were expressed with proportions. The association between 2 or more categorical variables was assessed using chi-squared and Fisher’s exact tests. Comparisons of 2 groups (D+DN with D-DN or healthy participants) based on HRQoL scores were performed using the independent t-test and reported as effect size (such as, mean difference) and 95% confidence interval (CI). Comparisons between 3 groups (D+DN, D-DN, and healthy participants) were carried out using analysis of variance with a post hoc Bonferroni test. Correlation of the participants’ HRQoL scores with NDS and NSS scores (not normally distributed) was evaluated using Spearman’s correlation (ρ). Multivariate analysis was carried out to determine factors that predict DN HRQoL, their coefficient, and 95% confidence intervals (CI) for factors associated with DN in patients with DM. We included new and previously researched covariates in the regression. A significant association between or among variables was declared if the *p*-value was <0.05.

## Results

### Sociodemographic characteristics of the participants

A total of 306 participants were recruited for the study. The participants were separately and matched for age and gender in three groups (D-N, D+N, and healthy participants from the source population) with 102 participants in each group. Forty-seven (46.1%) of the participants in each group were female, 94.1% were married, and 83.3% had formal education (Western or Islamic). All the participants were Saudi nationals.

The mean age of the participants was 54.1 ± 12.8 years. There was no significant difference among the groups in terms of age (*p*=0.957) and gender (*p*=1.0). Table 1 presents the distribution of the participants based on sociodemographic characteristics.

**Table 1:** - Socio-demographic characteristics of DM patients with and without DN and healthy participants, Bisha, Saudi Arabia.

Variables	Participants			*P*-value
	DM with DN	DM without DN	Healthy control	
Age	54.1±12.8	54.1±12.8	53.5±13.1	0.9752
***Gender***				
Male	53	53	53	1.000
Female	49	49	49	
***Occupation***				
Private	55	9	1	0.001
Public	22	93	101	
Others	25	0	0	
***Marital status***				
Married	99	93	89	0.036
Single	3	9	13	
***Education***				
Educated	88	82	73	0.033
Not educated	14	20	29	
***Financial satisfaction***				
Satisfied	92	98	89	0.080
Not satisfied	10	4	13	
***Smoking***				
Yes	14	11	17	0.475
No	88	91	85	

DN: diabetic neuropathy, DM: diabetes mellitus

### Diabetes-related characteristics of the participants

Diabetes mellitus was diagnosed in 204 participants (with and without DN). The median duration of diabetes was 6 years (IQR, 7 years). Of the patients with DM, 189 (92.7%) reported adherence to their diabetes medications. The mean BMI of patients with DM was 33.7 ± 5.2 kg/cm^2^ and their mean HbA1c was 7.5% ± 0.96%. Among the patients with DM, 18 (8.8%) had a history of nephropathy, 19 (9.3%) had a history of transient ischemic attack (TIA), 18 (8.8%) had a history of retinopathy, 97 (47.8%) had systemic hypertension, 6 (2.9%) had ulcers, 51 (25%) had some form of urinary incontinence, and 115 (56.4%) had dyslipidemia. Nerve conduction studies (NCS) were performed on 82 patients in the D+DN group; of these, 73 (89%) had abnormal NCS findings. There was no difference between the D+DN and the D-DN groups in the presence of nephropathy (*p*=0.082), TIA (*p*=1.000), retinopathy (*p*=0.082), hypertension (*p*=0.942), dyslipidemia (*p*=0.778), foot ulcers (*p*=0.407), urinary incontinence (*p*=0.628), diabetic medication adherence (*p*=0.7885), and physical inactivity (*p*=0.390; Table 2).

**Table 2:** - Comparison of diabetes-related characteristics across patients with and without diabetic neuropathy, Bisha, Saudi Arabia.

**Diabetes –related characteristics**	**No/Total**	**OR**	**95%CI of OR**	***P*** **-value**
***Nephropathy***				
Present	13/102	2.80	0.90, 10.52	0.048
Absent	5/102			
***TIA***				
Present	10/102	1.10	0.39,3.28	0.810
Absent	9/102			
***Retinopathy***				
Present	13/102	2.80	0.90, 10.52	0.048
Absent	5/102			
***Hypertension***				
Present	48/102	0.96	0 .54, 1.73	0.889
Absent	49/102			
***Dyslipidemia***				
Present	59/102	1.10	0.62,2.04	0.672
Absent	56/102			
Foot ulcers				
Present	4/102	2.00	0.28, 22.96	0.407
Absent	2/102			
***Urinary incontinence***				
Present	24/102	0.90	0.43,1.69	0.628
Absent	27/102			
***Adherence to medications***				
Present	94/102	0.90	0.26, 2.86	0.786
Absent	95/102			
***Physical inactivity***				
Present	94/102	0.60	0.15, 2.19	0.390
Absent	95/102			

OR: odds ratio, CI: confidence interval, TIA: transient ischemic attack

Health-related quality of lifein patients with diabetes with neuropathy compared with age- and gender-matched healthy participants

The patients with D+DN had poorer HRQoL mean scores compared with apparently healthy participants, both overall (*p*<0.001) and across all domains of the Norfolk QOL-DN, including the physical functioning/large fiber (*p*<0.001), ADLs (*p*<0.001), symptoms (*p*<0.001), small fiber (*p*<0.001), and autonomic (*p*<0.001) domains (Table 3).

**Table 3:** - Comparison of HRQoL by DM patients with and without DN, Bisha, Saudi Arabia.

**HRQoLdomains**	**Mean±SD**	**MD**	**95%CI of MD**	***P*** **-value**
***DM with DN versus DM without DN***				
**Physical functioning**				
DM with DN	31.6 ± 11.2	26.47	24.00, 28.96	<0.001
DM without DN	5.2 ± 6.2			
**ADLS**				
DM with DN	10.2 ± 6.2	10.00	8.77, 11.23	<0.001
DM without DN	0.2 ± 0.9			
**Symptoms**				
DM with DN	11.8 ± 4.9	9.81	8.80, 10.83	<0.001
DM without DN	2.0 ± 1.8			
**Small fibers**				
DM with DN	9.9 ± 4.1	9.28	8.38,10.17	<0.001
DM without DN	0.6 ± 1.9			
**Autonomic**				
DM with DN	7.2 ± 3.5	6.81	6.10,7.53	<0.001
DM without DN	0.4 ± 1.2			
**Overall**				
DM with DN	70.8 ± 24.8	62.37	57.21,67.53	<0.001
DM without DN	8.4 ± 9.2			
***DM with DN versus healthy population***				
**Physical functioning**				
DM with DN	31.6 ± 11.2	31.61	29.42, 33.79	<0.001
Healthy population	0.01 ± 0.02			
**ADLS**				
DM with DN	10.2 ± 6.2	10.23	9.01, 11.44	<0.001
Healthy population	0.095 ± 0			
**Symptoms**				
DM with DN	11.8 ± 4.9	9.24	8.12,10.35	<0.001
Healthy population	2.6 ± 2.9			
**Small fibers**				
DM with DN	9.9 ± 4.1	9.91	9.10, 0.72	<0.001
Healthy population	0.1 ± 0.02			
**Autonomic**				
DM with DN	70 ± 3.5	7.21	6.53, 7.89	<0.001
Healthy population	0.1 ± 0.01			
**Overall**				
DM with DN	70.8 ± 32	70.79	65.96, 75.63	<0.001
Healthy population	0.1 ± 0.03			

HRQoL: health-realted qulaity of life, MD: mean difference, DN: diabetic neuropathy, DM: diabetic mellitus, SD: standard deviation, ADLS: activities of daily living

Multiple comparisons of the 3 groups using analysis of variance and post hoc Bonferroni testing confirmed a significant difference among the 3 groups and between each combination of 2 groups across all HRQoL domains (*p*<0.001).

Health-related quality of life of patients with diabetes and neuropathy compared with age- and gender-matched patients with diabetes without neuropathy

Using the Norfolk QOL-DN, which comprises 5 domains and one overall HRQoL mean score, the D+DN group had significantly poorer HRQoL scores compared to the D-DN group in both the overall HRQoL score (*p*<0.001) and across all domains: physical functioning/large fiber (*p*<0.001), activities of daily living (ADLs) (*p*<0.001), symptoms (*p*<0.001), small fiber (*p*<0.001), and autonomic domains (*p*<0.001; Table 3). In the D+DN group, there was a strong correlation between NDS score and overall HRQoL score on the Norfolk QOL-DN (Spearman’s ρ= −0.71, *p*<0.001). Similarly, a strong correlation was found between NSS score and overall HRQoL score on the Norfolk QOL-DN (Spearman’s ρ= −0.81, *p*<0.001; Table 4). Summary statistics of the correlation matrix of BMI, duration of DM, HbA1C, and age with HRQoL score are also shown in Table 4.

**Table 4:** - Correlation between covariates and HRQoL among patients with DM and DN, Bisha, Saudi Arabia.

DN-QOL						
Variables	Total	Physical functioning	ADL	Symptoms	Small fibers	Autonomic
*NDS	ρ=0.71 *p*<0.001	ρ=0.64 *p*<0.001	ρ=0.60 *p*<0.001	ρ=-0.36 *p*=0.002	ρ=0.62 *p*<0.001	ρ=0.55 *p*<0.001
**NSS	ρ= 0.81 *p*<0.001	ρ=-0.79 *p*<0.001	ρ=0.85 *p*<0.001	ρ= 0.68 *p*<0.001	ρ=0.80*p*<0.001	ρ 0.80*p*<0.001
Body mass index	ρ=0.10 *p*=0.093	ρ= 0.09 *p*=0.119	ρ=0.16 *p*=0.005	ρ=-0.13 *p*=0.029	ρ=0.11 *p*-=0.061	ρ=0.15 *p*<0.008
Duration of DM	ρ=0.82 *p*<0.001	ρ=0.79 *p*<0.001	ρ=0.77 *p*<0.001	ρ= 0.75 *p*<0.001	ρ=0.76 *p*<0.001	ρ=0.73 *p*<0.001
Hemoglobin A1C	ρ=0.30 *p*<0.001	ρ=0.30 *p*<0.001	ρ=0.30 *p*<0.001	ρ= 0.22 *p*=0.001	ρ=0.27 *p*=0.001	ρ=0.26 *p*<0.001
Age	ρ=0.008 *p*=0.896	ρ=0.01 *p*=0.9317	ρ=0.01 *p*=0.936	ρ= 0.06 *p*=0.291	ρ=0.02 *p*=0.735	ρ=0.05 *p*=0.399

*NDS: neuropathy disability score, **NSS: neuropathy symptoms score HRQoL:health-realted qulaity of life, DN: diabetic neuropathy, DM: diabetic mellitus, ADL: activity of daily living

### Relationship between electrophysiology study findings and HRQoL

With the exception of the symptoms domain of the Norfolk QOL-DN, there were significant differences in mean HRQoL scores between participants with normal and abnormal NCS results (Table 5).

**Table 5:** - Relationship between NCS and HRQoL in DM patients with DN, Bisha, Saudi Arabia.

NCS(Abnormal=73 Normal=9)	Mean	MD	95%CI of MD	*P*-value
***All***				
Abnormal	78.89 (23.4)	19.45	3.01, 35.88	0.011
Normal	59.44 (23.3)			
***Physical functioning***				
Abnormal	35.14 (10.6)	9.69	2.27, 8.56	0.005
Normal	25.44 (9.7)			
***ADLS***				
Abnormal	12.19 (5.9)	4.41	0.31, 17.07	0.019
Normal	7.78 (6.0)			
***Symptoms***				
Abnormal	12.12 (5.9)	-0.76	-4.18, 2.65	0.672
Normal	12.89 (3.4)			
***Small fibers***				
Abnormal	11.23 (3.6)	3.68	1.08, 6.27	0.003
Normal	7.56 (4.1)			
***Autonomic***				
Abnormal	8.21 (3.1)			
Normal	5.78 (3.0)	2.43	0.23, 4.62	0.015

MD: mean difference, NCS: nerve conduction studies, ADLS: activities of daily living

### Predictors of HRQoL in patients with diabetes and neuropathy

On regression analysis, physical inactivity (*p*=0.043), duration of DM (*p*<0.001), abnormal NCS, NDS (*p*<0.001), and NSS (*p*<0.001) predicted HRQoL in patients in the D+DN group (Table 6). The regression model was a good fit of the data, F(21,44)=13.53, *p*<0.001, and our independent variables explained 86.6% of the variability in the overall HRQoL (R^2^=0.866).

**Table 6:** - Summary of Rosai-Dorfman disease cases from 2 tertiary hospitals in the Western regions of Saudi Arabia.

Variable	Coefficient	SE	t	95%CI	*P*-value
Age	-0.27	0.12	-0.22	-0.28, 0.22	0.827
Gender	-0.34	2.72	-0.12	-5.79,5.11	0.902
Education	-7.67	6.68	-1.15	-21.04,5.70	0.256
Marital status	-18.09	12.35	-1.46	-42.82,6.63	0.148
Body mass index	0.23	0.28	0.82	-0.33,0.78	0.413
Hemoglobin A1c	-1.24	1.61	-0.77	-4.46,1.98	0.444
Smoking	12.90	17.51	0.74	-22.14,47.95	0.464
Physical inactivity	-14.53	7.01	-2.07	-28.56, -0.50	0.043*
TIA	-10.57	9.30	-1.14	-29.18,8.03	0.260
Retinopathy	-3.33	14.98	-0.22	-33.32,26.66	0.825
Financial satisfaction	0.47	9.72	0.05	-18.99,19.92	0.962
Ulcers	-2.79	6.45	-0.43	-15.70,10.13	0.668
Urinary incontinence	-4.34	3.61	-1.20	-11.56, 2.88	0.234
Adherence	-4.18	5.32	-0.79	-14.82,6.47	0.435
Duration of DM	2.08	0.42	4.93	1.24, 2.93	0.001*
Neuropathy disability score	-6.10	1.38	-4.44	-8.86-3.35	0.001*
Neuropathy symptoms score	-1.71	0.85	-2.02	-3.40, -0.01	0.048*
Dyslipidemia	1.46	2.89	0.50	-4.32,7.24	0.616
NCS	13.09	5.24	2.5	2.32,23.86	0.019

HRQoL:health-realted qulaity of life, Ci: confidence interval, NCS: nerve conduction studies, DM: diabetes mellitus, DN: diabetic neuropathy, TIA: transient ischemic attack, SE: standard error

## Discussion

The present study compared HRQoL findings among D+DN, D-DN, and apparently healthy participants. Our results indicated a significant difference between D+DN and D-DN and between D+DN and healthy individuals in overall HRQoL scores and across the 5 domains of HRQoL on the Norfolk QOL-DN. This result is consistent with previous studies that focused on both DN pain specifically and DN in general.^21-23^ Our finding suggests that the perceptions of patients with DM and DN of their physical, mental, sexual, cognitive, self-perception, and social aspects of their lives and overall well-being are worse than the perceptions of both persons with DM without DN and apparently healthy individuals drawn from the same population. Given that the 3 groups in the study were well-matched for age and gender, and as our results showed no significant between-group differences in the other background characteristics such as hypertension, cardiovascular events, dyslipidemia, smoking, urinary issues, adherence to medications, physical inactivity, and foot ulcers, confounding factors were unlikely to influence the HRQoL assessment outcomes. However, it is possible that the effect of DN on HRQoL transcends the impact of the pain component of neuropathy, as previous studies have shown that DN in patients with DM independently affects the physical and mental components of HRQoL even after controlling for pain and pain severity.^24^

We observed a reduction in perceived physical functioning in the D+DN group. Physical functioning is a serious public health concern given that mobility impairments, such as compromised walking speeds and difficulty with safely negotiating the physical environment may impact an individual’s socioeconomic and mental well-being. Although common symptoms of DN, including numbness, paraesthesia, pain, and tingling are treatable, the adverse impact of DN on physical functioning may be experienced even before overt clinical manifestations occur.^25,26^ Hence, prevention of these complications and their associated impacts on physical function is critical.

Consistent with previous reports, this study found a significant correlation between NSS, NDS, and HRQoL scores in patients in the D+DN group.^21,27,28^ This result could be a reflection of the high levels of pain, paraesthesia, numbness, and cramping often reported by patients with DM and DN, which have an undeniable impact on their physical and mental well-being. In addition, in agreement with previous studies reporting that prolonged disease duration is a significant factor related to poor HRQoL in patients with DM,^22,24,29,30^ we found a significant correlation between duration of DM and HRQoL. Given the absence of a correlation between patient age and HRQoL, the present study has shown that the duration of DM is more consequential than age in determining HRQoL in patients with DM and DN. This finding increases the importance of prevention, as people who are diagnosed at a younger age will have a longer duration of illness over their lifetime and thus a higher risk of poorer HRQoL.

Our results confirm the reports from previous studies that revealed a relationship between patients’ HbA1C and HRQoL.^31,33^ This finding also corroborates the results of a longitudinal study that showed that a reduction in hyperglycemic symptoms resulted in an improved HRQOL.^34^ One of the targets of DM management is to improve glycemic control and reduce the risk of diabetes-associated complications, which, in turn, can improve DM patients’ HRQoL.^33^ To attain this target, there needs to be unwavering cooperation between patients and their attending physicians. Intuitively, patients’ compliance can be expected to improve when their HRQoL improves as a consequence of treatment adherence. Nonetheless, it should be noted that there have also been studies assessing the relationship between HRQoL and HbA1C or glycemic control that reported contrary findings in patients with type 2 DM.^35^ In our study, the presence of abnormal NCS findings was also significantly associated with impaired HRQoL in patients with DM and DN.

Our results demonstrated that 5 of the covariates considered-NDS, NSS, NCS result, physical inactivity, and duration of DM-independently predicted overall HRQoL. In previous studies, duration of DM has been reported to be an important predictor of HRQoL,^22^ although there is a paucity of data on the relationship between NDS or NSS and HRQoL.

While the aforementioned covariates were the most important predictors of impaired HRQoL in the D+DN group as determined by a disease-specific HRQoL measurement tool, in other studies employing different HRQoL tools with varying psychometric properties, MNSI, HbA1c, mental fatigue, depression, treatment, female gender, diabetic complications, non-diabetic comorbidities, and coronary artery disease have been found to be important predictors of HRQoL status.^2,22^

Although some previous studies have reported that BMI negatively influenced HRQoL scores,our study results, like many others,did not show a significant relationship between BMI and HRQoL.^2,22,27,29,36^ Of note, the strength of association between neuropathy or neuropathic pain and HRQoL was found to be dependent on both the type of HRQoL tool employed (such as, whether a disease-specific or generic HRQoL tool was used) and the HRQoL domains investigated.^28,37^ The Norfolk QOL-DN tool utilized in this study is reliable across different populations, with a high sensitivity profile for both small- and large-fiber nerve impairment.^13^

As demonstrated in the present study, DN has a significant negative effect on HRQoL, as the condition limits physical activity and interferes with ADLs. Therefore, we recommend that clinicians include regular assessment of HRQoL in their management plan for patients with DN. In addition, preventive strategies coupled with patient education should be considered key factors in the prevention and alleviation of morbidity and mortality rates.

### Implications of our findings and future research

We report a significant impact of DN on the HRQoL of participants in this study. Therefore, clinicians need to develop strategies and intervention programs aimed at promoting the health status of patients with DM from the time of diagnosis to preclude the progression of the disease and its complications. Doing so is critical given the undesirable effect of DN on quality of life and its negative impact on available therapeutic options. Evidence-based factors that are strongly associated with poor HRQoL will also be integral tools in the early identification of patients who may require intensive intervention as well as psychotherapy.

We suggest the use of a disease-specific HRQoL tool to gather important information regarding patients’ perceptions of their health, which may not be fully explained by the patients’ objective health status. This additional information may aid physicians in engaging patients in discussions concerning the impact of complications of DM, such as DN, on the overall disease outcome and achieving optimum disease control and patient well-being.

Future operational research can include larger samples drawn from multiple clinical sites to improve on the generalizability of our findings. Future studies can also explore potential interventions to generate the evidence-basis for effective measures to improve HRQoL especially those targeting persons with poor HRQoL scores.

A major study strength of the present study is the use of a disease-specific HRQoL instrument, which has been advocated for in previous studies and that employed a generic HRQoL outcome measure.^22,28^ In addition, the present study explored the relationship between electrophysiological findings and HRQoL in a cohort of diabetic patients with DN.

### Study limitations

First, this study was limited by its use of laboratory parameters that were obtained from medical records and therefore prone to the potential of incomplete data. This limitation, however, was mitigated by excluding records with missing data. Second, the study was cross-sectional, and as such, interpretation of causal relationships should be made with caution. Regardless of these limitations, this study constitutes one of the most comprehensive efforts to compare HRQoL scores in patients with DM and DN with age- and gender-matched patients with DM without DN and healthy participants.

In conclusion, a significant difference in the quality of life of participants with diabetes and diabetic neuropathy, compared to age- and gender-matched comparison groups of patients with dabetes and no DN, and healthy counterparts. In addition, NDS score, NSS score, physical inactivity, abnormal NCS findings, and longer duration of DM independently predicted poor HRQoL in patients with DM complicated by DN.
